# The health benefits of selenium in food animals: a review

**DOI:** 10.1186/s40104-022-00706-2

**Published:** 2022-05-13

**Authors:** Brittany M. Pecoraro, Diego F. Leal, Alba Frias-De-Diego, Matthew Browning, Jack Odle, Elisa Crisci

**Affiliations:** 1 College of Veterinary Medicine, Department of Population Health and Pathobiology, North Carolina State University, Raleigh, North Carolina USA; 2 Laboratory of Developmental Nutrition, Department of Animal Science, North Carolina State University, Raleigh, North Carolina USA

**Keywords:** Antimicrobial, Antioxidant, Food animals, Immunomodulation, Pig, Selenium

## Abstract

Selenium is an essential trace mineral important for the maintenance of homeostasis in animals and humans. It evinces a strong antioxidant, anti-inflammatory and potential antimicrobial capacity. Selenium biological function is primarily achieved by its presence in selenoproteins as a form of selenocysteine. Selenium deficiency may result in an array of health disorders, affecting many organs and systems; to prevent this, dietary supplementation, mainly in the forms of organic (i.e., selenomethionine and selenocysteine) inorganic (i.e., selenate and selenite) sources is used. In pigs as well as other food animals, dietary selenium supplementation has been used for improving growth performance, immune function, and meat quality. A substantial body of knowledge demonstrates that dietary selenium supplementation is positively associated with overall animal health especially due to its immunomodulatory activity and protection from oxidative damage. Selenium also possesses potential antiviral activity and this is achieved by protecting immune cells against oxidative damage and decreasing viral replication. In this review we endeavor to combine established and novel knowledge on the beneficial effects of dietary selenium supplementation, its antioxidant and immunomodulatory actions, and the putative antimicrobial effect thereof. Furthermore, our review demonstrates the gaps in knowledge pertaining to the use of selenium as an antiviral, underscoring the need for further in vivo and in vitro studies, particularly in pigs.

**Graphical abstract:**

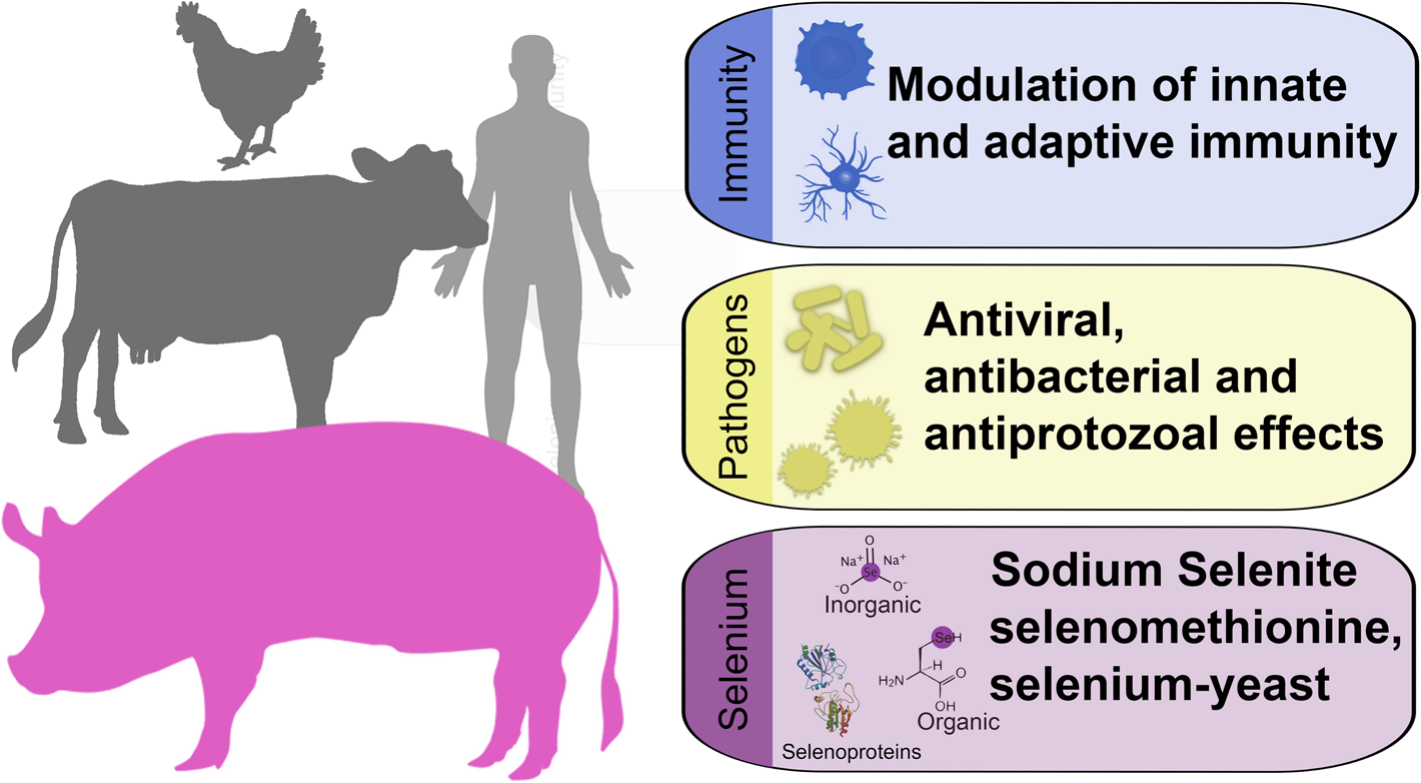

## Introduction

Selenium (Se) is an essential dietary trace element for animals and humans, playing indispensable roles in several physiological processes [1]. In nature Se exists mostly in two forms: Selenites with tetravalent (Se^4+^), and Selenates with hexavalent (Se^6+^) cations, respectively, from which all other selenium species are derived [2]. With respect to plants, Se is not an essential element but plants are able to convert mineral forms of Se present in the soil into various organic forms, namely selenomethionine and metylselenocysteine [3].

Se exhibits antioxidant, anti-inflammatory, anti-carcinogenic, potential antiviral and antibacterial activity [4]. Its biological function is achieved through the insertion of this trace element into a family of proteins known as selenoproteins [5]. Among these, glutathione peroxidase (GSH-Px) is a pivotal component of the antioxidant glutathione pathways which detoxifies lipid peroxides and provides protection of cellular and subcellular membranes against reactive oxygen species (ROS) damage [6]. It is worth mentioning that synthesis of selenoproteins is regulated by the availability of Se and, in situations of low availability, Se is supplied for synthesis of certain selenoproteins in detriment of others [7, 8].

Se exerts pronounced immune-modulatory capacity. Low levels of this trace mineral are linked to a debilitated immune system [9]. Likewise, Se is involved non-specifically in the immune response, being important for chemotactic and phagocyte activity and respiratory burst activities [4]. With regards to macrophage activity, Se mitigates the cytotoxic effects of ROS, reduces intracellular pathogen replication, enhances macrophage numbers and their phagocytic potential [8]. Adaptive immunity is also affected by selenium intake including the activation and functions of T- and B- cells [9, 10]. In the same context, inflammatory gene expression can be influenced by hyperoxidation, thus Se may also affect inflammatory response by regulating the oxidative state of immune cells [11].

Another characteristic of Se that has attracted much attention is its potential antiviral capacity. Oxidative stress is a hallmark of viral infections and, in many cases, ROS may contribute to increased viral replication, leading to an amplification loop [12]. Indeed, the increase of ROS production has been well-documented during human immunodeficiency virus (HIV), hepatitis B virus (HBV), hepatitis C virus (HCV), Epstein-Barr virus (EBV), herpes simplex type 1 (HSV-1), vesicular stomatitis virus (VSV), human T cell leukaemia virus type 1 (HTLV-1) and influenza viral infections [13]. Additionally, Se deficiency can alter chemokine and cytokine expression during viral infections [14]. Therefore, supplemental Se can enhance GSH-Px activity which protects the cell against free-radical oxidant injury [15].

Several studies have tested different sources of Se (i.e., organic and inorganic) against various pathogens (i.e., viruses, bacteria and protozoa; Table 1), showing general positive systemic benefits for a several species. Se supplementation suppressed TNF-α-induced HIV replication in culture [16]. Likewise, Se inhibits activation of HIV-1 in cell culture [17]. In pigs, supplemental Se inhibited porcine circovirus type 2 virus (PCV2) replication in PK-15 cells [15]. Further, mice subjected to herpes simplex virus 2 infection (HSV-2) presented decreased viral load in vaginal tissue and higher levels of tumor necrosis factor alpha (TNF-α) and interferon gamma (IFN-γ) [18].

**Table 1:** Summary of in vivo and in vitro studies evaluating the health benefits of Selenium

Source	Targeted species^a^	Pathogen	Effect	References
SeMet	Pigs	Virus	Antiviral	[15, 19]
Se-yeast	Humans	Virus	Antiviral	[16]
Se-yeast	Pigs	Bacteria	Antibacterial	[2]
Se-yeast	Ruminants	Bacteria	Antibacterial	[20]
SeMet; Se-yeast	Poultry	Bacteria	Antioxidant, immunomodulation, antibacterial	[21, 22]
Se-yeast; SeMet	Pigs	N/A	Immunomodulation, antioxidant	[23–30]
Sodium selenite	Humans	Virus	Antiviral	[31]
Se-yeast; Sodium selenite	Poultry	Virus	Antiviral	[32]
Se-enriched alfalfa	Ruminants	Bacteria	Immunomodulation	[33]
Sodium selenite	Poultry	Bacteria	Antibacterial	[34]
Se-enriched probiotics	Poultry	Protozoa	Antioxidant	[35]

*N/A* not applicable; the antimicrobial effect of Se was not evaluated in the study

^a^In some studies cell culture models were used

As Se supplementation into animal production has grown, the swine industry specifically has seen widespread economic and health benefits of its use [36]. Modern commercial pig production, based on high animal density in close quarters, has led to increased chances of infectious disease outbreaks along with associated mortality, increased control and treatment costs and decreased production values for the surviving pigs [37]. These outbreaks are a well-known cause of economic losses for the swine industry [38]. Furthermore, Se plays a leading role in the immune system; notwithstanding, the true extent of Se immunomodulatory capacity remains to be elucidated. Swine in vivo studies specifically pertaining to its use as an antiviral as well as a larger database of in vitro studies demonstrate the current gap of knowledge within this subject. Existing data (Table 2) underscores the need for further research, because more notably for pigs, supplemental dietary Se increases overall health, growth performance, meat quality, reproductive function and immune functionality, thereby reducing the burden caused by infectious diseases [37]. Moreover, due to similar selenoprotein profile, anatomic features and physiology to those of humans, pigs are an excellent model for translational research [39]; therefore, data arising from Se studies in pigs could be used to improve human health. This review will pinpoint historical and current knowledge on the beneficial outcomes of dietary selenium supplementation within food animals, with special attention given to pigs.

## Biological function of selenium and metabolism

Se is a metalloid trace element found in the environment as organic and inorganic forms. There are four different inorganic chemical forms; selenide (Se^2−^), elemental state selenium (Se^0^), selenite (Se^4+^), and selenate (Se^6+^). Se competes with sulphur (S) in biochemical pathways, and is incorporated into the S-containing amino acids, cysteine (Cys) and methionine (Met) forming organic Se compounds like selenocysteine (SeCys) and selenomethionine (SeMet) [1]. Studies have found that plants and grains absorb inorganic forms better, while organic forms are more efficiently metabolized by mammals [2, 53]. Selenocysteine is one such organic variant that is commonly found within animal sourced Se products, while enriched hay and cereals are common inorganic variants [54]. It is worth mentioning that Se exhibits synergy with vitamin E and is better absorbed in the presence of this vitamin [54], thus, diseases caused by Se deficiency are more severe when they occur concomitantly with vitamin E deficiency [55].

The absorption of Se occurs mainly in the duodenum. Absorption takes place primarily by active transport through a sodium pump. The mechanism of intestinal absorption of Se may differ depending of the chemical form of the element. Selenite is absorbed by simple diffusion; whereas, selenate is absorbed by a cotransport of sodium selenate and exchange selenate/OH^−^ [8]. Organic forms (i.e., selenomethionine and selenocysteine) follow the mechanisms of amino acid uptake. The ingested selenomethionine is absorbed in the small intestine by an active mechanism similar to that used for methionine uptake via intestinal methionine transporters [56] and enters the methionine pool of the body [57]. Selenomethionine can also be metabolized in the liver through the methionine cycle and transsulfuration pathways, yielding selenocysteine as a transient form, which is promptly converted into selenide, which in turn, is used for selenoprotein synthesis [8, 58]. Interestingly, in broilers, Se is mainly absorbed in the jejunum by a non-saturated diffusion process [59].

Selenocysteine (Sec) is a key component of selenoproteins. Sec is recognized as the 21^st^ amino acid [60]. Its incorporation into such proteins results from being co-translationally inserted during protein synthesis [24, 61]. Furthermore, the presence of selenocysteine rather than cysteine in the active site of an enzyme increases enzymatic efficacy up to 1000 fold [58]. Upon closer examination of Se on cellular activity, it is noted that nearly all selenoproteins are redox enzymes [62], which plays an influential role in immune cell signaling and function [21, 24] . GSH-Px is a family of selenoezymes, consisting of six isoforms (GPX 1–6) that presents selenocysteine on each subunit [63], serving as a shield for the cell to protect itself against hydrogen or lipid peroxides, which contribute to cardiovascular disorders, anemia, atherosclerosis, and inflammation [58, 63, 64]. Mammalian selenoproteins include: glutathione peroxidases (GPX 1–6), thioredoxin reductase (TR 1–3), iodothyronine deiodinases (D 1–3), selenophosphate synthetase (SPS2), and several thioredoxin-like selenoproteins, some of which serve as antioxidants within the body [65]. When selenoproteins function as antioxidants, they protect against cellular damage by stabilizing deleterious free radical molecules, which presents a pronounced influence in the immune system when it occurs within immunomodulatory organs such as the lymph nodes and spleen [37, 66].

The influence of Se supplementation also is reflected in its capacity to modify the expression and activity of more than 25 selenoproteins which are involved in oxidative stress, detoxification, transport mechanisms, metabolisms, and inflammatory responses [33, 65, 67].

For humans the recommended daily supplementation of Se is 55-75 μg/d. Within this dosing range, Se enhances the immune system, and stimulates a more efficient production of proteins and enzymes [6, 65, 68]. Larger doses as high as 200 μg/d are associated with the prevention of illnesses such as cancer, cardiovascular disease, and decreased viral mutation [69]. Of particular note, human dietary intakes range from high to low according to geography [70]. Indeed, Se levels in soil and food are low in some regions of China, New Zealand, and parts of Europe and Russia, making the recommended daily supplementation higher due to a lower average blood Se concentration in their populations [9, 71].

For food animals, an upper limit value for inorganic Se of 0.5 mg/kg and organic Se (Se yeast, L-SeMet) of 0.2 mg/kg of complete feed was established by The European commission [1] and 0.3 mg/kg by the FDA [72]. Notwithstanding, studies have shown that Se requirements for poultry may be much higher than the recommended upper limit [73–75]. Of particular interest, according to the NRC (2012) [76], the dietary Se requirements ranges from 0.3 ppm for weanling pigs to 0.15 ppm for finishing pigs; for gestating and lactating sows the dietary Se requirement is set at 0.15 ppm. Se concentrations in blood plasma are used to assess the efficacy of the Se dosage in supplemented feed. Plasma Se concentrations of ≥8 μg/dL are recognized as an adequate status in a healthy animal [40].

Se also plays a pivotal role within the gastrointestinal system through the connection between the gut microbiota and the host’s immune system, which has promoted the application of different forms of Se in the maintenance of gut immunity and microbiota [77]. Such supplementation stimulates the differentiation and proliferation of epithelial cells which regulate intestinal homeostasis and thus, the treatment of such an environment with dietary Se ultimately strengthens the host’s immune system and helps the host to tolerate the antigens naturally present within the gut [78].

Reported studies have shown that Se deficiency is followed by reduction of T cell counts, antibody responses, and neutrophil efficacy [79], decreasing the capacity to build a robust immune response and increasing the susceptibility to environmental challenges such as infection. Other health disorders derived from Se deficiency include Mulberry heart disease (MHD) and hepatosis dietetica (HD) in pigs [55], white muscle disease in calves [80], encephalomalacia, and exudative diathesis in chicks [81]. Correspondingly, in humans, Se deficiency results in a highly lethal cardiomyopathy known as Keshan disease [82]. Likewise, Se levels were found to be decreased in the brain of Alzheimer’s patients [83]. Low selenium concentration in plasma was associated with 4- to 5-fold increased risk of prostate cancer [84].

Even though numerous benefits to Se supplementation have been already described, the determination of an adequate dosage to achieve the best host responses without leading to toxicity is often a challenge. Excessive intake of Se results in toxicity (seleniosis). Common effects of selenosis in humans include the weakening and/or loss of hair and nails, mottling of teeth, nausea, and nerve damage [85]. Of particular note, an increased risk for type 2 diabetes has been reported in human subjects taking supplementary Se [86]. Evidence from animal studies demonstrated that elevating dietary Se intakes (0.4 to 3.0 mg/kg of diet) above the nutrient requirements, similar to overproduction of selenoproteins, led to insulin resistance and/or diabetes-like phenotypes in pigs [87]. One potential mechanism underlying the diabetogenic effect of Se could be due to elevated activity or expression of selenoproteins, resulting in over-scavenging of ROS, which in turn, leads to inhibition of protein tyrosine phosphatases and suppressed insulin signaling [87].

In livestock, symptoms of selenium intoxication commonly observed are hair loss, hoof deformities and reduced productivity [8], whereas acute exposure to high Se intake will result in death from respiratory failure [88]. Se nanoparticles are an alternative that have been considered to prevent toxicity and increase chemical stability and biocompatibility [89, 90]. Indeed, nano-Se results in higher Se retention and glutathione S-transferase activity due to its smaller size and higher bioavailability [91].

## Selenium in food animals

Se deficiency in food animals can markedly affect productive efficiency and health. Lower weight gains, reduced milk yield, and reduced fertility are among the effects of Se deficiency observed in livestock; furthermore, health problems particularly due to cell membrane damage, resulting from peroxides and immunosuppression, are also observed [92].

### Poultry

In poultry, Se supplementation exerts beneficial effects against several diseases, including coccidiosis, necrotic enteritis and pathogenic *E. coli*. Indeed, Se-enriched probiotics enhanced growth performance, antioxidant capacities, glutathione peroxidase-1, glutathione peroxidase-4 and IFN-γ mRNA gene expression, reduced oocysts shedding, and the cecal lesion scores of chickens and provided protection against *E. tenella* [35]. Dietary Se supplementation exerted a positive effect on body weight gain and feed efficiency of broilers reared under heat stress conditions [93]. In the same way, a significant increase of *Lactobacilli* spp. and *Bifidobacteria* spp., and a concomitant decrease of *Escherichia coli* and *Salmonella* spp. populations were observed in hens supplemented with organic Se [93]. Broiler chickens experimentally challenged with *E. maxima* and *Clostridium perfringens* and supplemented in ovo with sodium selenite had increased body weight, reduced intestinal lesions and oocyst production, increased levels of transcripts for interleukin-1-beta (IL-1β), interleukin-6 (IL-6) and interleukin-8 (IL-8) in intestine as well as increased serum antibody levels to *C. perfringens* α-toxin and NetB toxin [22]. Chickens inoculated with *E. coli* (serotype O1:K1) in the lower abdominal air sac had reduced mortality rate and air sac lesions when supplemented with inorganic Se [34]. Nano-Se supplementation improved antioxidant status and also increased IgG and IgM concentrations in chickens under oxidative stress [94]. Broilers supplemented with dietary Se had higher Se concentrations and GSH-Px activity in the liver, and higher serum antibody titer against H5N1 (Re-4 strain) [95]. Further, broilers fed Se-supplemented diets from 22 to 42 days of age had higher average daily feed intake and daily gain, and increased GSH-Px activity in breast and thigh muscles [96]. Of note, supplementation with Se-yeast was more effective than sodium selenite in improving meat quality of broilers [97]. Similarly, compared to sodium selenite, Se-yeast was more available for enhancing Se concentrations in plasma and tissues, and the expression and activity of GSH-Px in the pancreas of broilers [98]. It is worth mentioning that Se from ultrafine sodium selenite was more available to broilers than Se from sodium selenite in enhancing the GSH-Px mRNA expression in plasma, liver and pancreas of broilers [99].

Se deficiency can cause structural damage to the immune organs of chickens, which is manifested by decreased growth of the thymus and bursa of Fabricius [100]. It is worth emphasizing that for chickens and turkeys, Se requirements may be higher than previously estimated, and it has been proposed that Se should be increased to values above of the FDA limits [72–74]. Indeed, Liao et al. [101] demonstrated that the optimal dietary Se levels would be 0.36 mg/kg to support the full expression of selenoproteins in plasma, liver and kidney, and 0.46 mg/kg to support the full expression of selenoproteins in the pancreas of broilers from 1 to 21 days of age. Likewise, Wang et al. [96] reported that for broilers, between 22 to 42 days of age, Se requirements should be 0.49 mg/kg to achieve its maximum concentration, and 0.37 mg/kg for the full expression of selenoproteins in plasma and various tissues.

### Ruminants

As aforementioned, in domestic animals, Se deficiency will lead to immunosuppression, make animals prone to bacterial and viral infections, and compromise neutrophil activity, antibody production, proliferation of T and B cells as well as cytodestruction by T lymphocytes and NK cells [90, 102]. In ruminants, Se supplementation improved immune response by increasing neutrophil expression L-selectin, IL-8 receptor, and toll-like receptor 4 (TLR4) in sheep affected by Necrotic Pododermatitis, thereby contributing to a faster recovery [20, 103]. It is important to mention that Se deficiency in calves, lambs and dairy goat kids leads to a serious disease known as white muscle disease (WMD) or nutritional muscular dystrophy (NMD) [104]. This disease is manifested clinically by stiffness, weakness and recumbency [4]. In young animals, WMD can also cause cardiac injury which results in sudden death within the first weeks after birth [90].

Studies have been conducted incorporating Se enriched milk into the diets of calves through the use of Se enriched yeast, and selenomethionine [105, 106]. The milk enhanced by organic variants consistently increased the Se milk content as well as yielded enhanced immune capabilities for calves [105]. The use of enriched milk specifically has allowed for an effective readily available mode of Se administration, and has branched into the use of other enriched foods for animal consumption [107]. Likewise, calves immunized with J-5 *Escherichia coli* bacterin, which had access to Se-enriched hay around weaning, had higher antibody titers and a greater neutrophil total antioxidant potential. This resulted in lower mortality rates, as well as improved weight gain [33].

### Pigs

The data supporting the benefits of Se supplementation within swine production has been derived from a number of in vivo studies, summarized in Table 2, which examine the roles that Se plays in immunomodulation, pregnancy, toxicity, and the resulting health effects from environmental stressors [91, 92].

A recent study compared the effects of two different Se sources: organic (i.e., selenomethionine, SeMeth/ Se-methylselenocysteine, MeSeCys) vs. inorganic (i.e., sodium selenite, NaSe) on immune function, overall health, and meat quality. The resulting data revealed that the organic forms of Se yielded stronger immune responses, and higher Se concentrations within tissues as opposed to the inorganic form [38]. Moreover, serum concentrations of IgG, IgA, and IgM of organic Se-supplemented groups were higher when compared to the inorganic diets. MeSeCysalso increased gene expression of a number of liver and muscle selenoproteins. SeMet and MeSeCys demonstrated advanced capabilities to improve overall immune function [38]. Other studies comparing organic and inorganic sources of Se yield similar results, where organic sources were consistently shown to increase serum Se concentrations, GSH-Px activity, and antioxidant ability [25, 26, 47, 108].

Increased dietary Se intake in pregnant sows increased Se levels and antioxidant capacities in both the sow and the piglets, while decreasing the level of inflammatory factors [46]. Se levels within sow colostrum and milk can be increased significantly when sows are supplemented with organic sources [46, 51]. Interestingly, inorganic Se was found to be more biologically available for enhancing sow serum GSH-Px activity [51].

Pig diets can be contaminated by mycotoxins which lead to detrimental health effects. Deoxynivalenol (DON) is a mycotoxin that causes immunosuppression in pigs [41]. Se has the potential to counteract DON-induced immunosuppression in piglets and for this reason was regarded as promising treatment for DON-mediated toxicity [52].

Heat stress is a well-known factor the can negatively affect pig health. Piglets fed a Se-enriched probiotic diet and raised under heat stress were more capable of maintaining immune functions (increased T lymphocyte proliferation and IL-2 concentration), had higher selenoproteins synthesis, and higher antioxidant capacities compared to control piglets and also showed increased growth performance [109].

Weaning is one of the most stressful events in the life of a piglet. They are separated abruptly from the sow, transferred to an unfamiliar environment, mixed with unacquainted pigs, have to cope with new pathogens and food-associated antigens and instead of highly digestible and palatable sow’s milk, weaned pigs have to rely on a solid dry diet composed with less digestible proteins [27]. Therefore, the immediate post-weaning period is marked by a host of immune alterations such as up regulation of inflammatory cytokines and augmented concentration of acute phase proteins [110]. Furthermore, maintenance of redox balance is of paramount importance for effective immunity and health of the gut [111]. In this sense, dietary strategies that enhance endogenous antioxidant capacity in weanling pigs have been sought. Dietary supplementation with organic Se significantly improved growth performance, antioxidant ability (higher levels of serum GSH-Px) and plasma Se content of weaning piglets [92]. Piglets born from sows supplemented with organic Se had lower serum IL-1β, IL-6 when challenged with LPS after weaning [112]. Supplemental organic Se was effective in decreasing inflammation and oxidative stress in weaning piglets orally challenged with *Salmonella typhimurium* by inducing activity of the lymphocytes and expression of antioxidant enzymes [2].

As stated above, Se deficiency in pigs results in the lethal cardiomyopathy MHD (Mulberry heart disease). It is believed that reduced antioxidant activity arising from Se deficiency leads to MHD [1]. The underlying pathophysiological mechanism behind this myopathy is assumed to be a result of cell membrane damage by free radicals, which in turn, leads to augmented mitochondrial calcium influx and degeneration of muscle fibers [55]. Importantly, young pigs are the most susceptible to the disease as Se levels are lower in weanling pigs than in adults [55]. Indeed, 54% of piglets had Se levels below reference ranges at weaning [28].

**Table 2:** Summary of in vivo studies evaluating the health benefits of selenium in pigs

Source^a^	Analyzed effect	References
Organic	Immunomodulation, antioxidant	[28, 30, 40–44]
	Growth performance, Se tissue concentration	[25, 30, 45]
	Bioavailability	[46]
	Selenoprotein activity, gene expression	[30, 47]
Organic/inorganic	Antioxidant	[23, 26, 48, 49]
	Immunomodulation	[24, 41, 50]
	Growth performance, Se Tissue concentration	[26, 38, 41, 50–52]
	Selenoprotein activity, gene expression	[2, 24]

^a^Selenium sources were organized by their use in pig in vivo studies. The analysis took into account the nature of selenium (i.e., organic, inorganic or both), and the outcomes of its incorporation into the diet

## The imunomodulatory role of selenium in pigs

Several studies have evaluated Se as a modulatory agent of the immune system. Se is incorporated in pig diets to avert diseases arising from Se deficiencies as well as to combat various infections by utilizing its known immunomodulatory properties [113–115]. Cellular immune response, measured by in vitro lymphocyte response to mitogen stimulations, was lower in weanling pigs fed a vitamin E and Se deficient diet for 25 d [116]. In the same study, GSH-Px activity was lower in response to Se and vitamin E deficiency.

Notably, circulating concentration of Se is significantly decreased as a result of inflammatory disorders. Pigs challenged with LPS had 36.8% and 16.6% lower Se concentrations in serum and spleen, respectively, which was accompanied by a decrease in GSH-PX activity in serum, thymus, and lymph nodes [117]. These results raise the question: Does the Se requirement change during immune challenges?

High parity sows have decreased concentration of Se in colostrum and milk; therefore, litters born from older high-producing sows may present low Se status at birth and weaning. Additionally, transfer of Se from sows to piglets is limited [7]. In this sense, numerous studies have attempted to increase colostrum and milk Se concentration by supplementing the dam’s diet. In these studies, there was comparison of inorganic (selenite) and organic (Se-yeast) Se supplementation at different levels. Sows fed the organic Se source had a greater transfer of Se to the neonate, colostrum, milk, weaned pig, and sow tissues compared to sows supplemented an inorganic Se source [29]. However, when sows were fed organic and inorganic Se sources, Se from organic source was better transferred to colostrum and milk, and consequently to piglets, but no influence was observed for immunoglobulin concentration in colostrum and milk, and haptoglobin concentration [118].

Piglets fed an organic Se source had decreased serum concentrations of pro inflammatory cytokines (TNF-α, IL-1β, and IL-6) when exposed to oxidative stress. Moreover, the expression levels of TNF-α, IL-6, IL-1β, TLR4, and nuclear factor-κB (NF-κB) in the liver and thymus were downregulated in pigs fed organic Se and subjected to oxidative challenge [30]. Se deficiency in pigs resulted in spleen pathological changes, which were marked by a reduction of the white pulp volume and the number of splenic cord cells. These morphological changes were accompanied by increased levels of pro-inflammatory cytokines (IL-1β, IL-6, IL-8, IL-17 and TNF-α) and decreased levels of anti-inflammatory cytokines (IL-10 and IL-13) [119]. Likewise, maternal Se supplementation during gestation decreased the level of inflammation, autophagy and endoplasmic reticulum stress in the thymus and spleen of weaning piglets induced by the LPS challenge [42]. Supplementation of growing pigs with Se prevented the upregulation of inflammation-related genes, namely IL-6, IL-1β, TNF-α, IL-8, induced by heat stress in the jejunal mucosa and also exerted a protective effect against intestinal barrier disruption [43]. In the same way, gilts supplemented with organic Se had increased concentration of serum IL-2 and IgG, increased concentration of intestinal IgA, and decreased concentration of serum IL-6 [44].

## Selenium as a potential antimicrobial within food animals and humans

The nutritional status of the host is closely associated with its capacity to fight infections. Nutritional deficiency is now recognized to affect viral pathogenicity rather than to only compromise immune function. Thus, dietary Se deficiency that impairs an appropriate antioxidant response in the host can alter the viral genome such that a normally benign or mildly pathogenic virus can become highly virulent in situations of poor nutritional status combined with oxidative stress [11].

Se supplementation was tested against avian influenza, H9N2. Both organic (Se-enriched yeast), and inorganic (sodium selenite) forms of Se were used at doses of 0.3 and 0.15 mg/kg of feed. Both forms of Se significantly decreased viral shedding within the chicken with the organic form yielding the most effective results [32]. Dietary Se has also been tested against the parainfluenza virus within lambs focusing on the primary and secondary immune responses following the viral challenge. Following infection, the Se-supplemented lambs showed strengthened immune activity soon after being inoculated with the virus [120]. The supplementation with 100 μg Se/d significantly increase the number of total T cells and Th cells and a better virus clearance in humans subjects receiving a live attenuated polio virus vaccine [31].

The porcine circovirus type 2 (PCV2), is a single-stranded DNA virus, belonging to the Circoviridae family (Group II). PCV2 infection may lead to postweaning multisystemic wasting syndrome, which seriously impacts the pig industry [121]. Studies have been conducted to evaluate the potential antiviral effects of Se against PCV2. In an in vitro study, PCV2 replication was inhibited by selenomethionine (SeMet) at a high concentration (6 mmol/L) and the increase in PCV2 replication by oxidative stress was blocked by SeMet at physiological concentrations (2 or 4 mol) [19]. In another in vitro study using PK15 cells, the inclusion of Se at 2 or 4 μmol and selenoprotein S overexpression was capable of blocking the increase in PCV2 DNA copy number and infected cell numbers [122]. Likewise, DL-selenomethionine was shown to decrease PCV2 replication at concentrations of 4–16 μmol/L [14]. The underlying mechanism behind the inhibitive effect of DL-selenomethionine on PCV2 replication is thought to be mediated through enhanced activity of GSH-Px that protects the cell against free-radical oxidant injury [14]. Free radicals, such as H_2_O_2,_ are recognized to promote PCV2 replication [123]. Se inclusion inhibited H_2_O_2_-induced PCV2 replication promotion in vitro [124]. Such results support the hypothesis of antiviral potential in pigs; however, the lack of in vivo studies warrants further investigations on the potential role of Se as an antimicrobial agent, namely against viruses.

## Conclusion

The literature trends consistently demonstrate the effectiveness of Se dietary supplementation to maintain homeostasis of several metabolic processes in food animals. This is achieved especially through its participation as a co-factor in selenoproteins, protecting cell membranes against oxidative damage. This is of special importance for the maintenance of optimal immune activity which will render the host more capable to fight viral infections. Particularly to pigs, this subject warrants more research on the immunomodulatory and antiviral roles of Se using in vivo models and against other viruses that cause substantial losses for pig production.

## Data Availability

Not applicable.

## Abbreviations

GSH-Px: Glutathione peroxidase
ROS: Reactive oxygen species
HIV: Human immunodeficiency virus
HBV: Hepatitis B virus
HCV: Hepatitis C virus
EBV: Epstein-Barr virus
HSV-1: Herpes simplex type 1
VSV: Vesicular stomatitis virus
HTLV-1: Human T cell leukaemia virus type 1
PCV2: Porcine circovirus type 2
Sec: Selenocysteine
TNF-α: Tumor necrosis factor alpha
IFN-γ: Interferon-Gamma
IL-1β: Interleukin-1-beta
IL-6: Interleukin-6
IL-8: Interleukin-8
NK: Natural killer cells
TLR4: Toll-like receptor 4
SeMet: Selenomethionine
SeCys: Selenocysteine
